# Sex hormones, adiposity, and metabolic traits in men and women: a Mendelian randomisation study

**DOI:** 10.1530/EJE-21-0703

**Published:** 2022-01-20

**Authors:** Nellie Y Loh, Edward Humphreys, Fredrik Karpe, Jeremy W Tomlinson, Raymond Noordam, Constantinos Christodoulides

**Affiliations:** 1 Oxford Centre for Diabetes, Endocrinology and Metabolism, Radcliffe Department of Medicine, University of Oxford, Oxford, UK; 2 Nuffield Department of Population Health, University of Oxford, Oxford, UK; 3 NIHR Oxford Biomedical Research Centre, OUH Foundation Trust, Oxford, UK; 4 Department of Internal Medicine, Section of Gerontology and Geriatrics, Leiden University Medical Centre, Leiden, The Netherlands

## Abstract

**Objective:**

Epidemiological and clinical studies have highlighted important roles for sex hormones in the regulation of fat distribution and systemic metabolism. We investigated the bidirectional associations between bioavailable serum testosterone (BioT) in both sexes and oestradiol (E2) in men and adiposity and metabolic traits using Mendelian randomisation (MR).

**Design and Methods:**

As genetic instruments for sex hormones, we selected all the genome-wide significant, independent signals from a genome-wide association studies (GWAS) in up to 425 097 European ancestry UK Biobank participants. European population-specific, summary-level data for adiposity, metabolic, and blood pressure traits were obtained from the largest publicly available GWAS. Sex-specific, two-sample MR analyses were used to estimate the associations of sex hormones with these traits and vice versa.

**Results:**

In women, higher BioT was associated with obesity, upper-body fat distribution, and low HDL-cholesterol although, based on analyses modelling the sex hormone-binding globulin-independent effects of BioT, the last two associations might be indirect. Conversely, obesity and android fat distribution were associated with elevated serum BioT. In men, higher BioT was associated with lower hip circumference and lower fasting glucose. Reciprocally, obesity was associated with lower BioT and higher E2, while upper-body fat distribution and raised triglycerides were associated with lower E2.

**Conclusions:**

Adipose tissue and metabolic dysfunction are associated with deranged sex hormone levels in both sexes. In women, elevated BioT might be a cause of obesity. Conversely, in men, higher BioT appears to have beneficial effects on adiposity and glucose metabolism.

## Introduction

Body fat distribution is an independent risk factor for type 2 diabetes (T2D) and cardiovascular disease (CVD). While upper-body (android) fat accumulation, especially visceral adipose tissue (VAT), is associated with increased CVD risk due to deranged glucose and lipid metabolism, lower-body (gynoid) fat distribution provides protection against T2D and atherosclerosis (1). Compared to men, women generally have more fat mass (2). Additionally, premenopausal women typically have a greater proportion of subcutaneous AT (SAT), particularly in the gluteofemoral depot, while men generally accumulate more upper-body fat and have a higher proportion of VAT than women (2, 3). Sex differences in lipid, glycaemic, and blood pressure (BP) traits have also been described, with women having a lower CVD risk factor burden compared to men (4). Accordingly, men have an elevated risk of T2D, CVD, and all-cause mortality than women during middle age (3, 4).

Sex-dependent differences in fat mass and distribution are first observed during puberty (5), highlighting the importance of sex hormones in AT development and distribution. Reduced testosterone levels in men are also associated with increased upper-body fat accumulation and insulin resistance (IR) (6). However, data on testosterone replacement therapy are inconsistent in showing beneficial effects on regional adiposity (6). Similarly, a decline in oestrogen levels during menopause in women is associated with changes in fat distribution with an increase of upper-body and visceral fat mass and a reduction in gluteofemoral fat mass (7). Endogenous oestrogens (8) but not exogenous oestrogen administration (9) also appear to have beneficial effects in the regulation of adiposity, fat distribution, and CVD risk profile in men. Conversely, most observational data suggest that endogenous, physiological circulating testosterone levels are positively associated with android fat accumulation and IR in women (6). Consistent epidemiological findings have also been reported in women with hyperandrogenism secondary to polycystic ovary syndrome (PCOS) (6, 10, 11, 12, 13). Nonetheless, there are presently no definitive data that PCOS women have increased upper-body fat and in particular, VAT compared to non-hyperandrogenic controls (6, 10, 12, 13, 14). Similarly, clinical trial evidence of testosterone administration in women is insufficient to confirm the apparently harmful associations between higher testosterone and elevated cardiometabolic risk reported in observational studies (15, 16, 17).

Mendelian randomisation (MR) is an epidemiological tool using data from genetic studies to estimate the non-confounded relationships between exposures and outcomes (18, 19). Several previous MR studies investigated the associations of genetically instrumented sex hormones and anthropometric, metabolic, and cardiovascular traits (20, 21, 22, 23). These studies utilised small numbers of relatively weak genetic instruments and reported negative associations between genetically instrumented sex hormone-binding globulin (SHBG) levels and T2D risk, null effects of oestradiol (E2) and testosterone on CVD risk, and an inverse association between genetically predicted higher BMI and serum testosterone in men. More recently, an MR study using many instrumental variables derived from a large GWAS in the UK Biobank (UKBB) investigated the associations of genetically influenced sex hormone levels and T2D, glycaemic, and body composition phenotypes. It was shown that higher testosterone was associated with increased lean body mass and lower T2D risk in men (24). Conversely, in women, higher testosterone increased the risk of T2D and PCOS (24). Herein, we extended these findings by conducting a bidirectional MR study to determine the associations between genetically instrumented serum testosterone in both sexes and serum E2 in men and anthropometric, glycaemic, as well as lipid and BP traits and vice versa. Where the causal relationship between two related traits is unknown, bidirectional MR can be used to determine the causal direction(s) of effect using two independent sets of genetic variants found to be associated with each trait in different GWAS. Because most circulating testosterone is bound to SHBG and is consequently biologically inactive, we focused on non-SHGB-bound (bioavailable) testosterone (BioT).

## Methods

We conducted a bidirectional, two-sample MR study to investigate the relationships between circulating sex hormone levels and adiposity (BMI, waist-to-hip ratio adjusted for BMI (WHRadjBMI), BMI-adjusted waist (WCadjBMI) and hip (HIPadjBMI) circumferences), glycaemic (glucose, insulin), lipid (triglycerides, LDL-cholesterol, HDL-cholesterol), and BP (systolic, diastolic) traits. As genetic instruments for sex hormones, we selected all the genome-wide significant SNPs from a GWAS conducted in up to 425 097 participants of European descent from the UKBB (24). Collectively, these signals accounted for 14 and 12% of the heritability of serum BioT in women and men, respectively, and 2% of the heritability of serum E2 in men. While a GWAS of E2 levels has also been conducted in women (25), the UKBB mainly comprises of postmenopausal subjects (>75%). Additionally, the aforementioned study (25) only identified two independent signals in women both of which became non-significant after adjustment for SHBG and testosterone levels. Consequently, we opted not to pursue women-specific E2 analyses. Since the genetic architectures of circulating BioT were separate between sexes, analyses were conducted on sex-specific outcomes. Furthermore, given the sex-specific genetic correlations between BioT and SHBG (rg = −0.74 in women; rg = −0.05 in men) and between E2 and SHBG in men (rg = 0.19) (24), we undertook additional (cluster-filtered) analyses with genetic instruments modelling the effects of sex hormones independent of SHBG as previously identified via cluster analyses (24). Finally, we conducted unfiltered and cluster-filtered MR analyses after excluding variants with larger effects on outcome than the tested sex hormone trait (Steiger-filtering). For outcome data, European population-specific summary-level statistics for adiposity and metabolic traits were obtained from the largest publicly available GWAS (Supplementary Table 1, see section on supplementary materials given at the end of this article). For all the exposure instruments for which there was no direct match in the outcome datasets, we used proxy SNPs based on a LD cut-off of r2 > 0.5.

Because, no sex-stratified lipid and blood pressure GWAS have been reported to date, we also conducted new sex-specific GWAS in European ancestry participants from the UKBB using standard methodology (26) as detailed in the Supplementary Information, followed by one-sample MR. In this regard, two-sample MR methods can be reliably used for one-sample MR performed within large biobanks since they yield similar performance, in terms of bias and precision of the MR estimate, except for the MR-Egger sensitivity analysis (27).

To examine reverse causality, i.e. the potential causal effects of adiposity and metabolic traits on circulating sex hormone levels, we used genome-wide significant genetic instruments from the respective studies (Extended Tables 1). Additionally, we conducted one-sample MR studies using sex-specific genetic instruments for these traits derived from the GWAS we conducted in the UKBB (Extended Table 1). For outcome data, we utilised summary statistics from Ruth et al (24).

We employed the inverse-variance weighted (IVW) approach for two-sample MR analyses, with additional sensitivity analyses using MR-Egger, weighted-median, and MR-PRESSO (detailed in Supplementary Information). Analyses were conducted using the TwoSampleMR package (v0.5.6) implemented in R (v4.1.0) statistical software (28). Results were corrected for multiple testing with *P* ≤ 0.004 (0.05/12) considered significant. This cut-off is based on assessments of three hormone traits and four classes of cardiovascular risk factors (anthropometric, glycaemic, lipid, and blood pressure). Given the high correlation between some traits, this level of correction provides a balance between rigorous results and avoiding false negatives. A statistically significant IVW result coupled with directionally consistent associations from all three sensitivity analyses was considered as sufficient evidence to claim a causal effect.

## Results

### MR analyses in women

**Figure 1:**
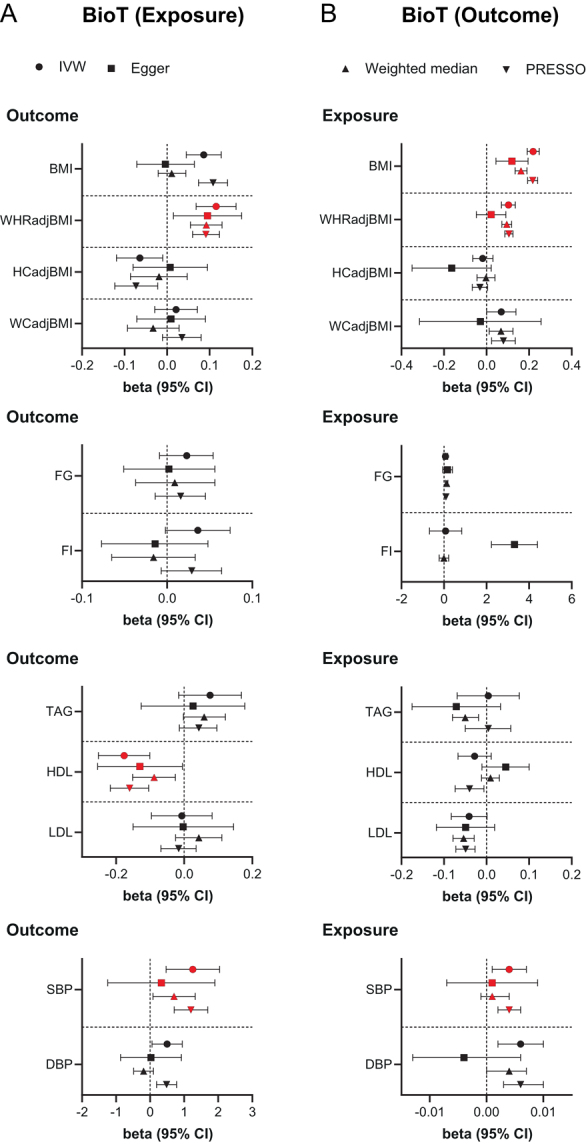
Bidirectional two-sample MR estimates of the relationship between bioavailable testosterone (BioT) levels and anthropometric, metabolic, and blood pressure traits in women. IVW, MR-Egger, weighted-median, and MR-PRESSO estimates (with 95% CI) are shown for primary analyses of (A) BioT on anthropometric, metabolic, and blood pressure traits (from Supplementary Table 2) and (B) anthropometric, metabolic, and blood pressure traits on BioT (from Supplementary Table 3). Red-filled symbols: analyses yielding IVW results with *P* ≤ 0.004 (Bonferroni correction for multiple testing) and that are directionally consistent across all sensitivity analyses. WHRadjBMI, BMI-adjusted waist-to-hip ratio; HIPadjBMI, BMI-adjusted hip circumference; WCadjBMI, BMI-adjusted waist circumference; FG, fasting glucose; FI, fasting insulin; TAG, triglycerides; HDL, HDL-cholesterol; LDL, LDL-cholesterol; SBP, systolic blood pressure; DBP, diastolic blood pressure.

Using IVW MR, elevated serum BioT was associated with higher BMI (β = 0.086 s.d., s.e. = 0.021, *P*  = 3.6 × 10^−5^), higher WHRadjBMI (β = 0.115 s.d., s.e. = 0.024, *P*  = 1.5 × 10^−6^), lower HDL-cholesterol (β = −0.176 s.d., s.e. = 0.039, *P*  = 4.8 × 10^−6^), and higher systolic BP (β = 1.254 mmHg, s.e. = 0.400, *P*  = 0.002) (Fig. 1A and Supplementary Table 2). Sensitivity analyses using weighted-median MR and MR-Egger (Fig. 1A and Supplementary Table 2) demonstrated uniform directions to IVW MR for all significant associations, except for that between BioT and BMI, which was directionally inconsistent in the MR-Egger model and was also associated with directional pleiotropy (MR-Egger intercept = 0.001). Comparable results were also obtained after Steiger-filtering to exclude variants with larger effects on outcome traits than BioT apart from the association between BioT and systolic BP, which became non-significant (Supplementary Table 2). In contrast, only a borderline significant association between elevated BioT and higher BMI persisted in MR analyses using the cluster-filtered genetic instrument, modelling the SHBG-independent effects of testosterone.

MR analyses in the reverse direction revealed that genetically determined higher BMI (β = 0.219 units, s.e. = 0.014, *P*  = 4.7 × 10^−53^) and higher WHRadjBMI (β = 0.103 units, s.e. = 0.016, *P*  = 4.3 × 10^−10^) were associated with elevated serum BioT in women (Fig. 1B and Supplementary Table 3). Higher systolic BP was also associated with raised serum BioT, but the effect size was minute (β = 0.004 units, s.e. = 0.001, *P*  = 0.004). Sensitivity analyses yielded similar results to IVW MR (Fig. 1B and Supplementary Table 3). However, they also provided evidence of unbalanced pleiotropy for the two main associations (p for pleiotropy from MR-Egger ≤ 0.01). Exclusion of pleiotropic variants, however, did not materially alter the findings using IVW regression (Supplementary Table 3). Similarly, MR analyses using the Steiger-filtered genetic instruments yielded consistent findings (Supplementary Table 3).

Since the lipid and BP GWAS summary statistics were derived from sex-combined populations, we also conducted, one-sample, bidirectional MR studies using women-specific data for these traits generated from the UKBB. These analyses yielded consistent results to the two-sample MR with sex-combined data (Supplementary Table 4). The effect size of genetically instrumented BioT on systolic BP, however, was an order of magnitude lower than in the primary analysis although, the association was strengthened (β = 0.120 mmHg, s.e. = 0.025, *P*  = 2.1 × 10^−6^) and remained significant after cluster and Steiger-filtering. The association between higher genetically predicted BioT and lower HDL-cholesterol also became robust to cluster-filtering.

Finally, because the instruments for the MR analyses involving sex hormones and BMI, WHRadjBMI, and BP traits were derived from partially overlapping populations, which may lead to biased estimates, we conducted additional MR analyses studies using summary statistics for anthropometric and BP traits excluding the UKBB cohort (Supplementary Table 5). These yielded directionally consistent IVW estimates, which were at least nominally significant in the unfiltered analyses for all the main, primary associations (Supplementary Table 5).

### MR analyses in men

**Figure 2:**
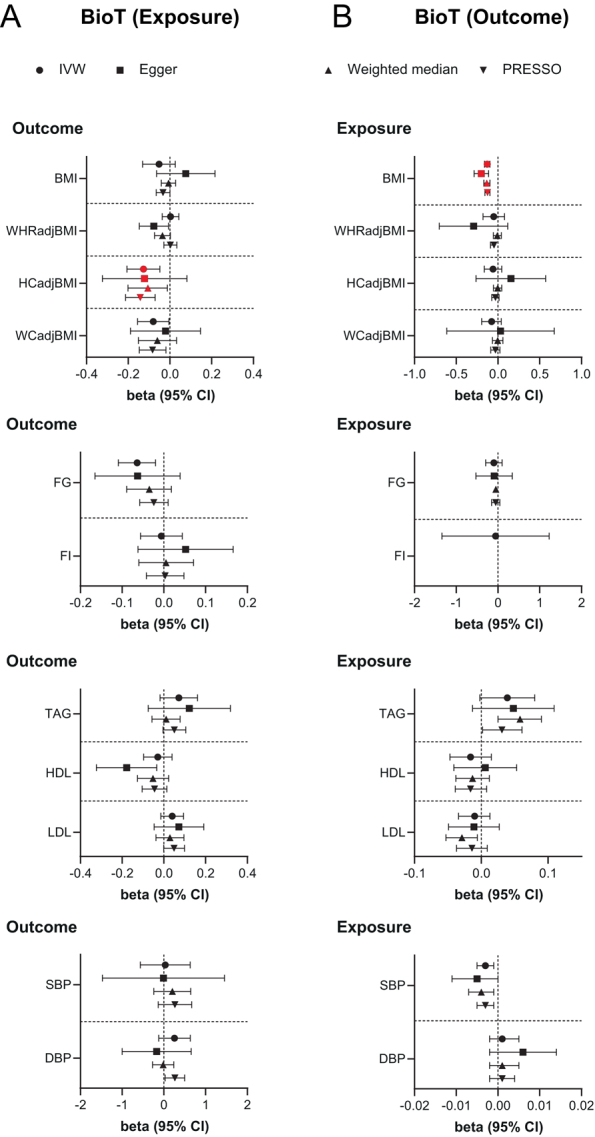
Bidirectional two-sample MR estimates of the relationship between bioavailable testosterone (BioT) levels and anthropometric, metabolic, and blood pressure traits in men. IVW, MR-Egger, weighted-median and MR-PRESSO estimates (with 95% CI) are shown for primary analyses of (A) BioT on anthropometric, metabolic, and blood pressure traits (from Supplementary Table 6) and (B) anthropometric, metabolic, and blood pressure traits on BioT (from Supplementary Table 8). Red-filled symbols: analyses yielding IVW results with *P* ≤ 0.004 (Bonferroni correction for multiple testing) and that are directionally consistent across all sensitivity analyses. WHRadjBMI, BMI-adjusted waist-to-hip ratio; HIPadjBMI, BMI-adjusted hip circumference; WCadjBMI, BMI-adjusted waist circumference; FG, fasting glucose; FI, fasting insulin; TAG, triglycerides; HDL, HDL-cholesterol; LDL, LDL-cholesterol; SBP, systolic blood pressure; DBP, diastolic blood pressure.

**Figure 3:**
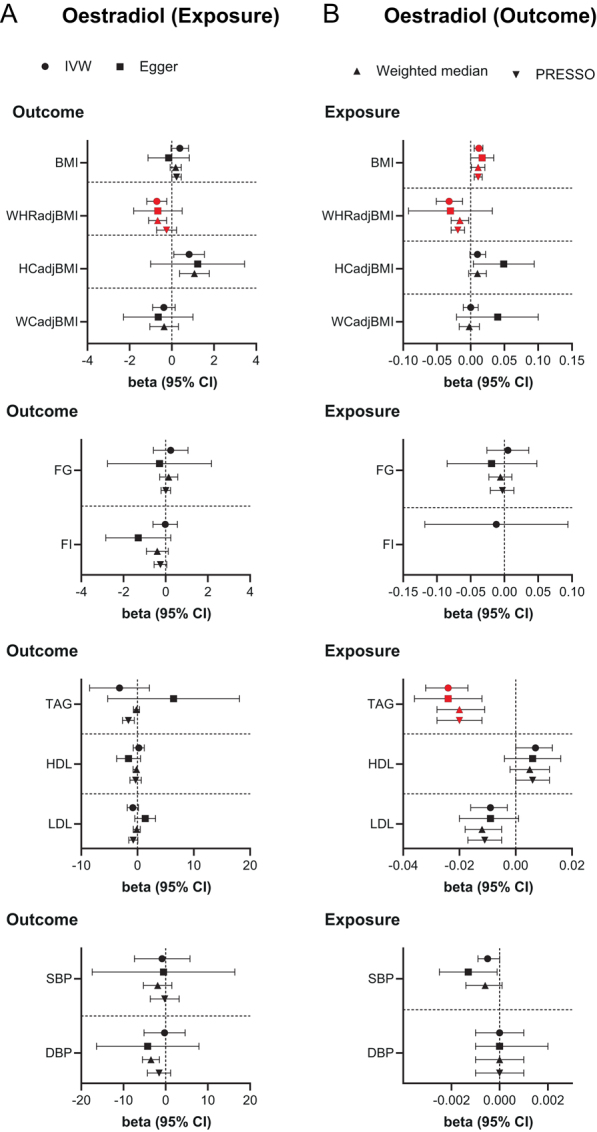
Bidirectional two-sample MR estimates of the relationship between oestradiol and anthropometric, metabolic, and blood pressure traits in men. IVW, MR-Egger, weighted-median, and MR-PRESSO estimates (with 95% CI) are shown for primary analyses of (A) oestradiol on anthropometric, metabolic, and blood pressure traits (from Supplementary Table 7) and (B) anthropometric, metabolic, and blood pressure traits on oestradiol (from Supplementary Table 9). Red-filled symbols: analyses yielding IVW results with *P* ≤ 0.004 (Bonferroni correction for multiple testing) and that are directionally consistent across all sensitivity analyses. WHRadjBMI, BMI-adjusted waist-to-hip ratio; HIPadjBMI, BMI-adjusted hip circumference; WCadjBMI, BMI-adjusted waist circumference; FG, fasting glucose; FI, fasting insulin; TAG, triglycerides; HDL, HDL-cholesterol; LDL, LDL-cholesterol; SBP, systolic blood pressure; DBP, diastolic blood pressure.

In men, genetically predicted elevated BioT was associated with lower HIPadjBMI (β = −0.127 s.d., s.e. = 0.040, *P*  = 0.002) and a trend for lower blood glucose (β = −0.064 mmol/L, s.e. = 0.023, *P*  = 0.005) (Fig. 2A and Supplementary Table 6), while higher genetically predicted serum E2 was associated with lower WHRadjBMI (β = −0.710 s.d., s.e. = 0.241, *P*  = 0.003) (Fig. 3A and Supplementary Table 7). Similar results were obtained in sensitivity analyses (Supplementary Tables 6 and 7). Furthermore, directionally consistent and robust associations were derived using the cluster-filtered and Steiger-filtered sex hormone exposure instruments (Supplementary Tables 6 and 7) apart from the association between E2 and WHRadjBMI, which was nominally significant in the cluster-filtered analysis and non-significant after Steiger-filtering (Supplementary Table 7).

MR analyses in the reverse direction revealed that higher BMI was associated with lower serum BioT (β = −0.128 units, s.e. = 0.017, *P*  = 9.9 × 10^−15^) (Fig. 2B and Supplementary Table 8) and higher circulating E2 (β = 0.012 units, s.e. = 0.003, *P*  = 0.0003) (Fig. 3B and Supplementary Table 9) respectively. Conversely, higher WHRadjBMI (β = −0.032 units, s.e. = 0.010, *P*  = 0.001) and elevated triglycerides (β = −0.024 units, s.e. = 0.004, *P*  = 1.1 × 10^−9^) were associated with lower E2 levels (Fig. 3B and Supplementary Table 9). Sensitivity analyses demonstrated consistent directions and similar effect estimates to IVW MR (Figs 2B, 3B and Supplementary Tables 8, 9). Congruent associations were also detected following Steiger-filtering (Supplementary Tables 8 and 9).

Finally, we conducted one-sample, bidirectional MR analyses between serum sex hormone levels and lipid and BP traits using male-specific data from the UKBB, which yielded directionally similar results to those obtained with two-sample MR with sex-combined data (Supplementary Table 10). Bidirectional MR analyses of sex hormones and BMI, WHRadjBMI, and BP traits using non-overlapping samples also yielded directionally consistent IVW estimates, which were at least nominally significant in the unfiltered analyses for all the main, primary associations (Supplementary Table 11).

## Discussion

Our study reveals that genetically instrumented higher BioT is associated with adverse effects on fat distribution and lipid profile in women. These results extend the MR findings of Ruth et al (24), which provided suggestive evidence of a causal link between higher BioT and elevated WHRadjBMI using anthropometric data from a smaller GWAS (29). They also complement results from observational studies, which have generally reported positive associations between higher androgen levels and central obesity, CVD risk burden, and T2D risk in women (11, 12, 13, 30, 31, 32, 33, 34). The mechanisms accounting for these links are unclear. Exposure to chronic androgen excess in women with PCOS was shown to suppress SAT lipolysis in some studies (13, 35, 36) and to impair SAT lipoprotein lipase activity, which controls the delivery of fatty acids from circulating triglyceride-rich lipoproteins to AT (13). Additionally, androgens were shown to inhibit adipogenesis of human adipose progenitors (APs)* in vitro* although this effect was independent of the sex or depot of origin of these cells (6). On the other hand, our cluster-filtered analyses suggest that the aforementioned associations might be indirect and driven primarily by SHBG, which may have functions beyond transporting sex steroids in blood and regulating their bioavailability and accessibility to target tissues (37). Indeed, ectopic over-expression of SHBG was shown to protect both female and male mice from genetic and diet-induced hepatosteatosis by directly reducing the production of key lipogenic enzymes in the liver (38). Additionally, SHBG treatment had anti-inflammatory and lipolytic effects on adipocytes and macrophages* in vitro* (39). Finally, we demonstrate a potentially direct causal link between raised serum BioT and obesity in women, which should be interpreted with caution since it was inconsistent across different MR models and was associated with directional pleiotropy. The latter may be driven by instrumental variables being associated with inflammatory bowel disease, diuretic use, birth weight, and sleep duration. Ruth *et al.* also provided suggestive evidence for such an association (24). Furthermore, a meta-analysis of randomised control trials (RCTs) found that testosterone treatment resulted in postmenopausal weight gain (16).

MR analyses in the reverse direction supported an effect of obesity in driving hyperandrogenism in women. Potential sources of the horizontal pleiotropy detected for this association include effects of genetic variants on fat distribution, tendency to perform vigorous physical activity, ovarian cancer risk, and thyroid status. Similarly, observational studies have highlighted an association between raised BMI and elevated testosterone levels in women (34) and worsening of hyperandrogenism in PCOS subjects (40). This link is thought to be partly driven by the IR, which accompanies excess adiposity. Accordingly, genetic syndromes of severe IR frequently present with marked ovarian hyperandrogenism (41). Mechanistically, hyperinsulinaemia stimulates androgen production from the ovarian theca cells both directly and by increasing pituitary luteinising hormone secretion (30, 42). Insulin also raises BioT levels by suppressing hepatic SHBG production (34, 42, 43, 44). Unfortunately, we were unable to lend support for the role of hyperinsulinaemia in linking obesity and hyperandrogenism due to a weak insulin exposure instrument (Supplementary Table 3). AT is also capable of androgen generation and might contribute to the higher BioT levels associated with obesity. Specifically, the androgen-activating enzyme aldoketoreductase type 1C3 (AKR1C3), generates testosterone from the androgenic precursor androstenedione, is abundantly expressed in AT and its SAT expression is induced in obesity, PCOS, and hyperinsulinaemia (35, 45). Indeed, increased SAT AKR1C3 activity was shown to contribute to circulating androgen levels in PCOS women (35). We further show that genetically instrumented android fat distribution is also associated with raised BioT, with direct effects of genetic variants on insulin resistance, circulating leptin levels, and reproductive tract tumour susceptibility potentially accounting for the horizontal pleiotropy detected for this link. The causal association between higher WHRadjBMI and hyperandrogenism is likely partly due to the IR associated with central obesity. The conversion of androstenedione to 5α-reduced androgens in cultures of SAT and VAT APs was also shown to be ten-fold greater than the formation of oestrone (46). Additionally, SAT *AKR1C3* expression was increased in women with android fat distribution (47).

In contrast to women, genetically instrumented raised BioT in men was associated with decreased HIPadjBMI and lower fasting blood glucose with cluster-filtering demonstrating that these associations are likely to be driven directly by testosterone. However, no associations were detected with other metabolic or BP traits. These results are congruent with previous MR studies in the UKBB showing positive links between higher BioT and increased lean mass (24, 48), reduced fat mass (48), and lower glucose (24). However, they are only partly consistent with observational data demonstrating inverse associations between circulating testosterone and risk of metabolic syndrome in men (31, 32, 49, 50, 51), as well as clinical findings in men with physiologic or chemically induced hypogonadism, who were shown to be at increased risk of developing obesity and IR, as well as T2DM (8, 52, 53). On the other hand, our data are aligned with RCTs of testosterone therapy in men, which reported benefits on adiposity as reflected by lower HIPadjBMI herein, as well as diminished fasting glucose levels and T2D risk (54, 55). However, in contrast to our findings, a meta-analysis of these trials also showed a reduction in IR and in hypogonadal men, a decrease in triglycerides and LDL cholesterol (54). These discrepancies may be due to threshold effects of testosterone on adiposity and metabolic traits with only hypogonadal men adversely affected (6, 49). Alternatively, SHBG might also have direct effects on adiposity and metabolic traits in men. Finally, because most (~85%) circulating oestrogens in men are generated from androgens via the peripheral activity of aromatase (8), it is possible that some of the beneficial effects of testosterone may be due to conversion to E2. Consistent with this, a RCT demonstrated that the positive actions of short-term testosterone replacement on adiposity in men were driven by E2 conversion (56). Furthermore, men with congenital oestrogen deficiency due to mutations in the aromatase gene or oestrogen resistance due to oestrogen receptor loss-of-function mutations develop central obesity and hyperinsulinaemia, which, in the case of aromatase deficiency, are corrected by oestrogen replacement (57, 58, 59). Accordingly, we provide tentative evidence that genetically predicted higher E2 levels in men are associated with lower-body fat distribution. However, this finding should be cautiously interpreted as it was not robust to cluster or Steiger-filtering, which might be due to a paucity of genetic instruments in the filtered analyses. Similarly, we did not detect causal effects of E2 on metabolic traits probably because the SNPs used as instrumental variables only explained 2% of the total variation in systemic E2 levels in men (24).

While in women, obesity was associated with hyperandrogenism, genetically instrumented raised BMI in men was causally linked to low BioT and raised E2 levels. In this regard, there is a well-established epidemiological relationship between both obesity/weight gain and hypogonadism in men, typified by low testosterone and high E2, which is corrected after weight loss (34, 53, 60, 61). Mechanistically, AT is enriched among metabolic tissues in *CYP19A1* expression (https://gtexportal.org), encoding aromatase and is a major source of E2 production in men. Furthermore, CYP19A1 transcript levels and activity are amplified in obesity (62, 63). Increased peripheral aromatisation of testosterone in obese men may also lead to enhanced central E2 signalling that suppresses gonadotropin production thereby exacerbating hypogonadism (8, 53, 64). Raised cortisol levels due to hypothalamo–pituitary–adrenal axis hyperactivity, systemic inflammation, hyperleptinaemia, and IR might also contribute to hypogonadism in obese men via central or testicular actions (34, 53). Finally, we found negative associations between genetically instrumented WHRadjBMI and hypertriglyceridaemia and E2 levels. Regarding the former, expression of CYP19A1 was shown to be lower in s.c. abdominal than gluteal AT (65). Furthermore, conversion of androgen precursors to oestrone was ten-fold higher in gluteal vs s.c. abdominal or VAT-derived APs (46). The mechanistic link between raised triglycerides and low E2 levels is unclear but might partly involve suppressed aromatase activity given that hypertriglyceridaemia was also nominally associated with raised BioT in men (Supplementary Table 7).

Our study has limitations. First, an assumption of MR is that the instrumental variables do not affect the outcome except through exposure. This issue was minimised through the use of multiple genetic variants, undertaking several sensitivity analyses and performing analyses with Steiger-filtering. Secondly, we did not employ recently developed MR methods like causal analysis using summary effect estimates (CAUSE) (66) that reduce the chances of false positives while accounting for sample overlap or Latent Heritable Confounder MR, which simultaneously estimates bidirectional causal effects and heritable confounding from GWAS summary statistics (https://wp.unil.ch/sgg/lhc-mr/). Thirdly, the summary statistics for glycaemic traits were generated from small GWAS and explained only a small percentage of the total variance in these traits. This may account for the lack of associations between sex hormones and fasting insulin in the MR analyses and vice versa. Fourthly, our findings are based on GWAS conducted in European ancestry participants and hence may not extend to other ethnic populations. Finally, our data relate to lifelong exposure to sex hormones and thus may not be synonymous with the shorter-term effects of hormone replacement therapy.

In summary, we provide evidence for a bidirectional interplay between sex hormones and metabolic and adiposity traits in men and women. Our data further demonstrate that high BioT levels in women may be both a cause and consequence of obesity. In contrast, the links between hyperandrogenism and upper-body fat distribution and dyslipidaemia might be indirect and driven by lower SHBG levels. Finally, we highlight a potentially vicious cycle in men, whereby low BioT and E2 levels cause AT and metabolic dysfunction, which in turn exacerbate hypogonadism. Future experimental studies should investigate the mechanistic bases for these links.

## Supplementary Material

Supplementary Materials

Supplementary Table 1. GWAS summary statistics used in Mendelian Randomisation study

Supplementary Table 2. Two-sample MR estimates of effects of BioT in women on anthropometric, metabolic and blood pressure traits.

Supplementary Table 3. Two-sample MR estimates of effects of anthropometric, metabolic and blood pressure traits on BioT (nmol/L) in women.

Supplementary Table 4. Bidirectional MR estimates of the relationship between BioT, and metabolic and blood pressure traits in women in the UKBB.

Supplementary Table 5. Bidirectional two-sample MR estimates of effects of BioT in women on anthropometric and blood pressure traits using UKBB-independent outcome datasets.

Supplementary Table 6. Two-sample MR estimates of effects of BioT in men on anthropometric, metabolic and blood pressure traits.

Supplementary Table 7. Two-sample MR estimates of effects of oestradiol in men on anthropometric, metabolic and blood pressure traits.

Supplementary Table 8. Two-sample MR estimates of effects of anthropometric, metabolic and blood pressure traits on BioT (nmol/L) in men.

Supplementary Table 9. Two-sample MR estimates of effects of anthropometric, metabolic and blood pressure traits on oestradiol (binary) in men.

Supplementary Table 10. Bidirectional MR estimates of the relationship between of BioT and oestradiol levels, and metabolic and blood pressure traits in men in the UKBB.

Supplementary Table 11. Bidirectional two-sample MR estimates of effects of BioT and oestradiol in men on anthropometric and blood pressure traits using UKBB-independent outcome datasets.

## Declaration of interest

The authors declare that there is no conflict of interest that could be perceived as prejudicing the impartiality of this study.

## Funding

C C is funded by a British Heart Foundationhttp://dx.doi.org/10.13039/501100000274 Clinical Research Fellowship (FS/16/45/32359). F K is funded by a British Heart Foundationhttp://dx.doi.org/10.13039/501100000274 Programme Grant (PG/12/78/29862). J W T is supported by the Medical Research Council http://dx.doi.org/10.13039/501100000265 UK (Programme Grant MR/P011462/1).
