# Supplementary material for: Sex hormones, adiposity, and metabolic traits in men and women: a Mendelian randomisation study
Source: Eur J Endocrinol. 2022 Jan 20;186(3):407–16. doi: 10.1530/EJE-21-0703 (PMC8859921; doi:10.1530/EJE-21-0703)
Supplement: Supplementary Materials [file supplementary_material.pdf]

Supplementary Information

Sex hormones, adiposity and metabolic traits in men and women: a Mendelian Randomisation study.

Nellie Y. Loh, Edward Humphreys, Fredrik Karpe, Jeremy W. Tomlinson, Raymond Noordam, and Constantinos Christodoulides.

Supplementary Methods

Exposure and outcome datasets for lipid and BP traits from the UK Biobank (UKBB)

The UKBB cohort is a prospective cohort with 502,628 participants between the age of 40 and 69 years recruited from the general population at multiple assessment centers across the UK between 2006 and 2010 (1). Invitation letters were sent to eligible adults registered to the National Health Services (NHS) and living within a 25 miles distance from one of the assessment centers. Participants provided information on their lifestyle and medical history through touch-screen questionnaires and physical measurements. Blood samples were collected for serum and plasma chemistry, and for genome-wide genotyping. The UKBB study was approved by the North-West Multicenter Research Ethics Committee (MREC). Access for information to invite participants was approved by the Patient Information Advisory Group (PIAG) from England and Wales. All participants provided electronic written informed consent for the study.

Sex-specific genome-wide association analyses for lipid and BP traits were performed on individuals of European descent using linear mixed models implemented in the program BOLT_LMM (version 2.3.2) (2). LDL-cholesterol was corrected for the use of lipid lowering agents by dividing the measured level by 0.7. In case of systolic blood pressure, 15 mmHg was added in case of use of blood pressure lowering medication. In case of diastolic blood pressure, 10 mmHg was added in case of use of blood pressure lowering medication. LDL-cholesterol, HDL-cholesterol, triglycerides, and systolic and diastolic BP were rank-based standardised (mean 0, SD = 1). We adjusted the analyses for age and the first 10

principal components, and corrected for the genetic correlation matrix (to correct for familial relationships in the UK Biobank population). Analyses were done on the autosomal chromosomes only. SNPs with a minor allele frequency <0.01 were excluded as well as SNPs with an imputation quality <0.3.

To select a set of independent SNPs for each trait as genetic instruments for MR analyses, we selected all variants with P_BOLT_LMM <5e-8 and performed LD clumping using ld_clump with the default set at LD $r^2 < 0.001$ and a genetic distance of 10Mb.

Mendelian Randomisation

We employed the inverse-variance weighted (IVW) approach for two-sample MR analyses, which assumes that all instruments included in the analyses are valid, affect the outcome only through the exposure, and do not associate with any confounders. However, since this is often not true, especially when using many genetic instruments, we performed sensitivity analyses including MR-Egger regression, weighted-median estimator, and MR-PRESSO (MR Pleiotropy RESidual Sum and Outlier). MR-Egger does not force the regression line through the intercept and is therefore able to test for the presence of directional pleiotropy. The weighted-median estimator assumes that at least 50% of the genetic instruments are valid (3). MR-PRESSO detects the presence of variant effect sizes that are outliers and corrects pleiotropy via outlier removal (4). Additional MR analyses were performed following Steiger filtering, a step where variants with larger effect sizes on outcome traits than on exposure traits are excluded from analyses. Analyses were conducted

using the TwoSampleMR package (v0.5.6) implemented in R (v4.1.0) statistical software (5).

Supplementary references

1. Sudlow C, Gallacher J, Allen N, Beral V, Burton P, Danesh J, Downey P, Elliott P, Green J, Landray M*, et al.* UK biobank: an open access resource for identifying the causes of a wide range of complex diseases of middle and old age. *PLoS Med* 2015 **12** e1001779.

2. Loh PR, Tucker G, Bulik-Sullivan BK, Vilhjalmsson BJ, Finucane HK, Salem RM, Chasman DI, Ridker PM, Neale BM, Berger B*, et al.* Efficient Bayesian mixed-model analysis increases association power in large cohorts. *Nat Genet* 2015 **47** 284-90.

3. Bowden J, Davey Smith G & Burgess S. Mendelian randomization with invalid instruments: effect estimation and bias detection through Egger regression. *Int J Epidemiol* 2015 **44** 512-25.

4. Verbanck M, Chen CY, Neale B & Do R. Detection of widespread horizontal pleiotropy in causal relationships inferred from Mendelian randomization between complex traits and diseases. *Nat Genet* 2018 **50** 693-8.

5. Hemani G, Zheng J, Elsworth B, Wade KH, Haberland V, Baird D, Laurin C, Burgess S, Bowden J, Langdon R*, et al.* The MR-Base platform supports systematic causal inference across the human phenome. *Elife* 2018 **7**.
